# Ancient pangenomic origins of noncanonical NLR genes underlying the recent evolutionary rescue of a staple crop

**DOI:** 10.1126/sciadv.ady1667

**Published:** 2025-10-10

**Authors:** Carl J. VanGessel, Terry J. Felderhoff, Daniil M. Prigozhin, Meihua Cui, Gael Pressoir, Adam L. Healey, John T. Lovell, Vamsi J. Nalam, Marc T. Nishimura, Geoffrey P. Morris

**Affiliations:** ^1^ Department of Soil and Crop Science, Colorado State University, Fort Collins, CO 80526, USA.; ^2^ Department of Agronomy, Kansas State University, Manhattan, KS 66502, USA.; ^3^ Berkeley Center for Structural Biology, Molecular Biophysics and Integrated Bioimaging Division, Lawrence Berkeley National Laboratory, Berkeley, CA 94720, USA.; ^4^ Department of Agricultural Biology, Colorado State University, Fort Collins, CO 80526, USA.; ^5^ CHIBAS, Centre Haitien d’Innovation en Biotechnologies pour une Agriculture Soutenable, #13 Rue EDH Lathan, Croix des Bouquets, Haiti.; ^6^ Genome Sequencing Center, HudsonAlpha Institute for Biotechnology, Huntsville, AL 35806, USA.; ^7^ Department of Biology, Colorado State University, Fort Collins, CO 80523, USA.

## Abstract

The recent adaptation of the cereal crop sorghum to a global aphid outbreak was a fortuitous case of evolutionary rescue, but the pangenomic and molecular basis is not known. We show that *RMES1* disrupts phloem feeding via activation of conserved immunity networks, with a growth-to-defense transition mediated by phytohormone signaling and activated by nucleotide-binding site–leucine-rich repeat receptor (NLR) resistance genes on a structural variant. The causative NLRs [resistance to *Melanaphis sorghi* 1A (RMES1A) and RMES1B] lack signaling domains and have adenosine triphosphatase mutations expected to abrogate function, suggesting that RMES1 NLRs regulate immunity via a noncanonical mechanism. The *RMES1* NLR family is ancient, orthologous to phloem-feeding resistance genes in rice and syntenic across the grass superpangenome. Thus, gene birth-and-death processes at an ancient gene cluster created rare standing variation and provided the adaptive allele for evolutionary rescue.

## Teaser

Ancient pangenomic origins of immunity genes underlie the recent evolutionary rescue of sorghum from an aphid invasion.

## INTRODUCTION

Evolutionary rescue occurs when populations in changing environments avoid extinction by rapid adaptation (*1*, *2*). Evolutionary rescue is expected to be driven by Mendelian traits encoded by single genes that undergo a selective sweep, whereas oligogenic or polygenic architecture may not be able to fix resilience quickly enough (*3*). In plants, molecular machinery responsible for evolutionary rescue has been described for flowering time pathways (*4*, *5*). Conserved pathways such as photoperiodism can be modulated for evolutionary rescue by loss of function of canonical flowering time genes (*5*). However, few studies have examined how evolutionary rescue manifests in the context of plant resistance to insect herbivores, particularly through immune receptor–mediated defense responses. Investigating the biological components of evolutionary rescue could facilitate adaptation to global environmental change. The recent adaptation of a staple monocot cereal crop sorghum [*Sorghum bicolor* (L.) Moench] following a global aphid outbreak provides an opportunity to characterize the pangenomic and molecular basis of a case of in situ evolutionary rescue (*6*).

Plants adapt to diverse pests via constitutive defenses or induced immunity (*7*). Some defense compounds are natively expressed to deter feeding directly such as glucosinolates, benzoxazinoids, and cyanogenic glucosides (*8*–*10*). In contrast, induction of common phytohormone and defense networks can result from recognition for various pests (*11*, *12*). *Resistance* (*R*) genes include intracellular receptor proteins, which detect molecular patterns and activate defense pathways through initial oxidative and Ca^2+^ bursts, subsequent phytohormone signaling, and ultimately developmental and physiological changes that affect fecundity and/or behavior (*13*, *14*). Intracellular *R* proteins are typically encoded by nucleotide-binding site (NBS)–leucine-rich repeat (LRR) receptor genes (NLRs), which directly or indirectly recognize pathogens in the plant cytoplasm (*15*). The NBS and LRR domains of NLRs function to inducibly form oligomeric “resistosomes” (*16*–*18*). In monocots, NLRs typically have an N-terminal coiled-coil (CC) domain, which forms a pentameric calcium channel to activate immunity (*19*). NLRs providing resistance to piercing-sucking herbivores include *Vat*, *Mi-1.2*, *Adnr1*, *BPH6*, *BPH30*, and *BPH40*; however, the extent of NLR diversity has not been fully characterized (*20*–*24*).

Aphids (Hemiptera: Aphididae) feed from the phloem of plants and are one of the fastest colonizing pests (*25*, *26*). Sorghum aphid (*Melanaphis sorghi* Theobald)) (*27*) emerged in 2013 in the Western Hemisphere as a major threat to sorghum that quickly spread to most growing regions in the Americas (*28*–*30*). The *Resistance to M. sorghi 1* locus (*RMES1*) on chromosome 6 (Chr06) was shown to underlie the evolutionary rescue of global sorghum production and in commercial breeding programs across the Americas after the aphid outbreak (*6*, *31*). *RMES1* is widely deployed for sorghum aphid resistance; however, the selection pressure placed on aphid populations highlights the potential for future biotype shifts (*32*). Competing hypotheses on the molecular basis of aphid resistance have been proposed involving cyanogenic toxicity or *R* gene–induced defenses (*6*, *31*, *32*). The cyanogenic glucoside dhurrin is a deterrent of chewing insects and the detoxification gene β-cyanoalanine synthase (*CAS*) is within the *RMES1* sweep region (*6*, *33*, *34*). However, LRR-encoding genes (referred to hereon as NLRs) are located within the original mapping interval (*31*). Here, we characterize the pangenomic origins and molecular basis of *RMES1*, describing extensive structural variation at a noncanonical NLR gene cluster that led to activated conserved immunity pathways necessary for evolutionary rescue.

## RESULTS

### *RMES1* disrupts aphid feeding to reduce fecundity

**Fig. 1.**
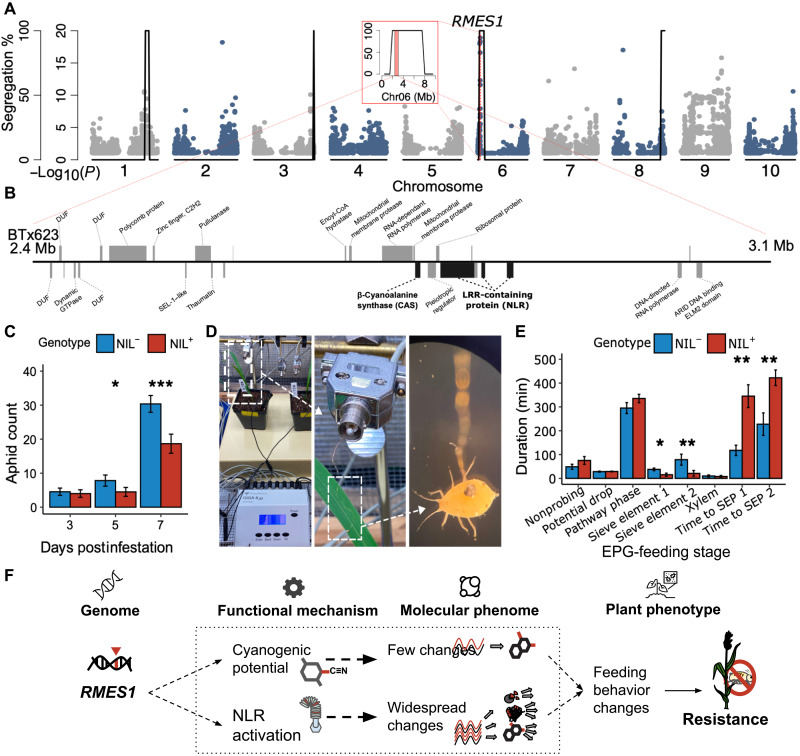
RMES1 reduces aphid fitness by disrupting feeding, but the molecular mechanism and pangenomic basis are unknown. (**A**) Selection scan (*F*_ST_) of Haitian breeding population and a global diversity panel (*6*) overlaid with NIL introgression plot identifies *RMES1* on the RTx430v2 (recurrent parent) sorghum genome. Outer axis shows NIL segregation between NIL^−^ (RTx430 recurrent parent haplotype) and NIL^+^ (donor haplotype). Inner axis shows significance [−log_10_(*P*)] of selection scan. Inset highlights Chr06 *RMES1* introgression and QTL from fixation scan as the red-shaded region (*6*). (**B**) Genes within *RMES1* QTL [red region of (A) inset] of BTx623v5 highlighting candidate causal genes. Genes above are in the forward orientation, and those below are in the reverse orientation. Black genes are functional candidates. Unlabeled genes were not assigned automated annotations. GTPase, guanosine triphosphatase; CoA, coenzyme A; DUF, domain of unknown function; ARID, A–T Rich interaction domain. (**C**) No-choice assay over 7-day infestation determining aphid fecundity. *P < 0.05 and ***P < 0.001. (**D**) Electrical penetration graph (EPG) experimental setup for detecting feeding behavioral changes. White dashed lines zoom to insect electrode on sorghum leaf and gold wire connected to aphid. (**E**) EPG duration spent at each feeding phase. SEP, sieve element phase. *P < 0.05 and **P < 0.01. (**F**) Competing functional and molecular hypotheses based on *RMES1* resistance.

The *RMES1* locus was first mapped in a biparental population to Chr06 (*31*), and a separate analysis of a Haitian breeding population under strong aphid infestation identified a selective sweep at *RMES1* (Fig. 1A) (*6*). To test mechanistic hypotheses, we developed BC_3_ near-isogenic lines (NIL^+^ and NIL^−^) with IRAT204 (*RMES1*^+^ donor) and RTx430 (recurrent parent), which were 94.7% isogenic (Fig. 1A). NIL^+^ contained a 5.16-Mb introgression from IRAT204 on Chr06 from 2.13 to 7.28 Mb (RTx430v2 reference). The syntenic region on the pangenome reference PI276837 (having the resistant *RMES1* single-nucleotide polymorphism S06_02892438) corresponds to 2.06 to 7.25 Mb (PI276837v1) and encompasses the *RMES1* quantitative trait locus (QTL) at ~2.78 to 3.21 Mb (PI276837v1; Fig. 1B) (*6*, *31*).

To confirm the previously reported antibiosis resistance trait, we used a no-choice assay to determine aphid fecundity on NILs (Fig. 1C) (*32*). At 5 days post infestation (dpi), *M. sorghi* populations were significantly lower in NIL^+^ plants compared to NIL^−^ (means ± SE, 4.2 ± 1.3 versus 7.8 ± 1.6; *t* test, *P* < 0.01). At 7 dpi, this effect was stronger (17.1 ± 2.4 versus 30.4 ± 2.4; *P* < 2 × 10^−4^). Because NLR-encoding regions from *Medicago truncatula* inhibit feeding of *Acyrthosiphon kondoi* (*35*–*37*), we next considered whether the *RMES1*-mediated fecundity reduction in sorghum aphid is due to altered feeding behavior (Fig. 1D and data S1). The electrical penetration graph (EPG) technique revealed aphids spent significantly less time in the phloem salivation (E1) and ingestion (E2) phases on NIL^+^ plants (Fig. 1E). Salivation time (E1) was nearly twice as long on NIL^−^ plants (*P* = 0.02), while phloem ingestion (E2) lasted approximately four times longer on NIL^−^ plants (*P* = 0.02). In addition, aphids had greater difficulty locating sieve elements on NIL^+^ plants, taking three times longer to initiate salivation and twice as long to begin ingestion. These results establish that *RMES1* disrupts aphid feeding behavior and reduces fecundity.

### *RMES1* response to infestation causes widespread molecular remodeling

On the basis of the predicted function of genes at the locus, cyanide detoxification via cyanoalanine synthase and induced immunity via NLRs were determined to be the top candidate mechanisms (Fig. 1, B and F). Analysis of the molecular response of uninfested (control) NILs and NILs infested for 24 and 48 hours post infestation (HPI) found a total of 4306 detected metabolites, of which 202 that were significantly affected by genotype or treatment (fold change > 2, *P* < 0.01). Cyanogenic glucosides, including dhurrin, were not differentially accumulated in the metabolome (data S2). Global expression changes were simultaneously determined using RTx430v2, and 26,120 of 34,601 genes were expressed. Genes regulating the dhurrin pathway were generally down-regulated after infestation in NIL^+^ while being unresponsive NIL^−^ (*33*) (fig. S1). Notably, the *CAS* gene at *RMES1* was not differentially expressed in all comparisons, and the biosynthetic steps were down-regulated in NIL^+^. This supports molecular adjustments in response to aphid infestation but not a hydrogen cyanide potential mechanism via *CAS*.

**Fig. 2.**
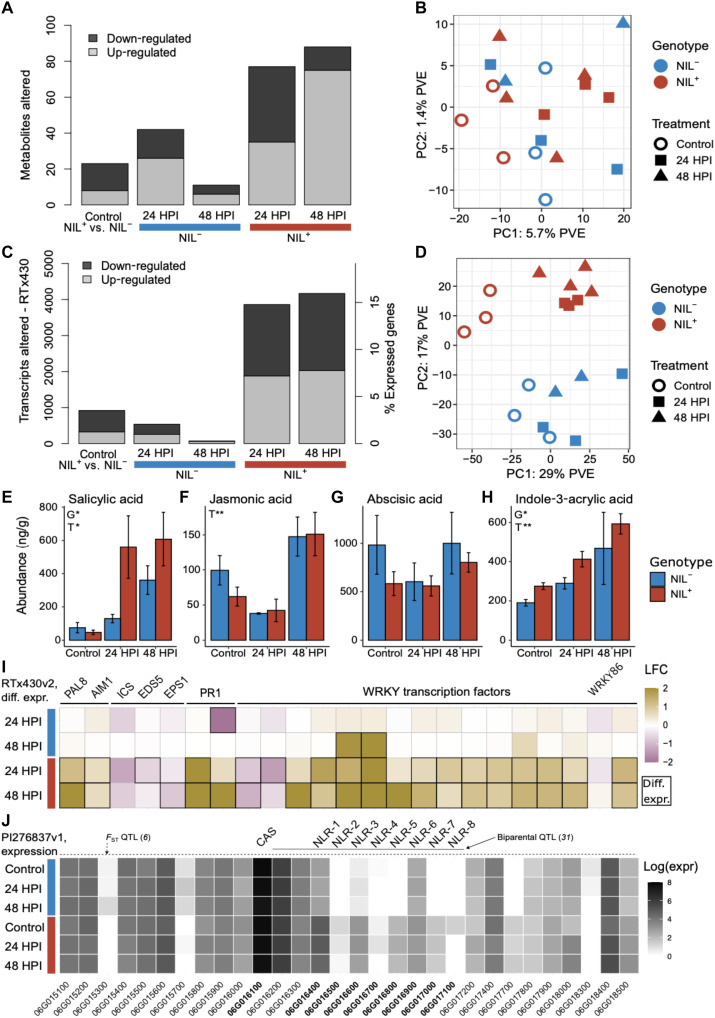
RMES1 broadly remodels the transcriptome and metabolome in response to infestation. (**A**) Differential expression of sorghum metabolomes during aphid infestation identifying *RMES1*-mediated changes (*P* < 0.01, fold change > 2). Control metabolites up-regulated in NIL^+^ relative to NIL^−^. NIL^+^/^−^ 24 and 48 HPI are up-regulated in each treatment relative to uninfested controls. (**B**) Principal components analysis (PCA) of metabolomes. PVE, percent variance explained. (**C**) Differential expression of global transcriptome changes during infestation of NILs. Expression was identified using the RTx430v2 recurrent parent for mapping (*P* < 0.05, fold change > 1.5). (**D**) Principal components analysis of transcriptomes. (**E** to **H**) Phytohormone abundance of NILs. Analysis of variance (ANOVA) genotype (G) and treatment (T) effects (*P < 0.05 and **P < 0.01). (**I**) Defense pathway transcriptional induction using RTx430v2 transcriptomes. SA marker genes and WRKY transcription factors are shown. Outlined boxes indicate differential expression relative to control. LFC, log fold change. (**J**) Log-transformed normalized expression of genes at *RMES1* using the PI276837v1 reference. CAS and NLR candidates are labeled above. Note that all gene identifiers are abbreviated from SbPI276837 IDs (e.g., SbPI276837.06G016400 to 06G016400).

NLR activation typically results in widespread changes to the metabolome and transcriptome, which was observed in NIL^+^ (Fig. 2A) (*38*). Overall, the metabolome principal component 1 (PC1) distinguished infested samples for NIL^+^ but not NIL^−^ (Fig. 2B). Metabolite functional classes were predicted, and diverse phenolic compounds were among those up-regulated in NIL^+^ relative to NIL^−^ (fig. S2). In response to infestation, hydrolyzable tannins, lignin-related compounds, and flavonoid glycosides were among up-regulated metabolites in infested NIL^+^ (fig. S3). Fewer metabolites responded in NIL^−^ than in NIL^+^. The diversity of functional classes responsive to infestation suggests that multiple metabolic pathways are activated by *RMES1*. Likewise, the transcriptome of infested NILs showed strong evidence of global immunity activation. Over 15% of expressed genes in NIL^+^ were differentially regulated (*n* = 8030, adjusted *P* < 0.05, fold change > 1.5) compared with 2% in NIL^−^ (*n* = 610) (Fig. 2C). PC1 captured transcriptional differences in infested NILs to a higher degree in NIL^+^ (Fig. 2D). PC3 distinguished time points at 24 and 48 HPI with uninfested samples intermediate (fig. S4). Differential expression and structure of metabolomes and transcriptomes indicate global-induced responses in an *RMES1*-dependent manner.

We next looked for evidence of defense networks contributing to molecular perturbations and found functional gene enrichment supporting NLR activation. The largest response in coexpression (MEturquoise, *n* = 2835) was down-regulation after infestation, with NIL^+^ having a stronger response than NIL^−^ (fig. S5). This module was enriched for photosynthesis and primary metabolic terms (data S3). Two modules with continued up-regulation over 24 and 48 HPI in NIL^+^ (MEblue and MEyellow) were enriched for response and signal transduction, cell redox homeostasis, and l-phenylalanine metabolic process terms. Overall, the signaling and metabolic responses are consistent with NLR-based immunity activation and suggest a growth-to-defense transition.

### *RMES1* activates multiple defense signaling pathways

To determine which phytohormones contribute to defense signaling, we quantified salicylic acid (SA), jasmonic acid (JA), 12-oxo-phytodienoic acid (12-OPDA), abscisic acid (ABA), and several auxin conjugates. SA, required for NLR-based *Mi-1* aphid resistance, had the strongest response and increased by ~10-fold (Fig. 2, E to H) (*21*, *39*). Phytohormones more abundant in NIL^+^ were SA [analysis of variance (ANOVA), *P* < 0.05], indole-3-acrylic acid (IACA; *P* < 0.05), and indole-3-aspartic acid (*P* < 0.01) (fig. S6). JA and its precursor in the jasmonate pathway, 12-OPDA, decreased at 24 HPI in both genotypes before rising, indicating a common role in early immunity activation unregulated by *RMES1*. This suggests that the causal mechanism at *RMES1* is inducing defense to aphids through SA signaling.

To confirm the activation of phytohormones signaling by *RMES1*, we assessed marker genes for significant responses (adjusted *P* < 0.05, fold change >1.5) (data S4). For SA biosynthesis, the phenylalanine ammonia-lyase (PAL) pathway was up-regulated (*PAL8* and *AIM1*), whereas the isochorismate synthase (ICS) pathway was down-regulated (*ICS*, *EDS5*, and *EPS1*) (Fig. 2I) (*40*, *41*). Signaling involved *PR1* homologs up-regulated in a genotype-treatment interaction (Fig. 2I) (*42*). Biosynthesis marker genes for oxylipins including JA, which plays dichotomous roles in sorghum aphid defense signaling (*43*), were differentially regulated in NIL^+^ (fig. S7). The related 9-lipoxygenase genes are associated with herbivory defense via death acid biosynthesis (*44*, *45*) and significantly up-regulated in NIL^+^ (*LOX1*, *LOX3*, and *LOX4*). WRKY transcription factors play diverse roles in response to environmental stressors, and *WRKY86* is the candidate gene underlying *RMES2* aphid resistance (*46*). Despite a strong general response by other WRKY genes, *WRKY86* did not significantly respond to aphid infestation and was slightly down-regulated at 24 HPI before up-regulating in both genotypes (Fig. 2I and fig. S8). The transcriptional activation of SA and oxylipin pathways, as well as WRKY genes, indicates that phytohormones and defense networks are activated by *RMES1*.

To investigate candidate genes with improved genome resolution, we mapped transcriptomes to the *RMES1*^+^ PI276837. Of the eight tandem NLRs in the resistant haplotype, five had negligible expression (<5 raw reads) in NIL^−^, suggestive of presence/absence variation (NLR-2, NLR-4, NLR-5, NLR-7, and NLR-8) (Fig. 2J). Three NLRs had a significant genotype effect (NLR-1, NLR-3, and NLR-5) (data S4). A putative C2H2-type zinc finger and thaumatin protein (SbPI276837.06G015200 and SbPI276837.06G015500) had a significant genotype and interaction effect; however, these fell outside of the biparentally mapped QTL (*31*). The differential expression of NLRs suggest that the strongest candidates for *RMES1* are NLR-1 (SbPI276837.06G016400), NLR-3 (SbPI276837.06G016600), and/or NLR-5 (SbPI276837.06G016800).

### A structural variant at *RMES1* harbors high copy number variation and causal NLRs

**Fig. 3.**
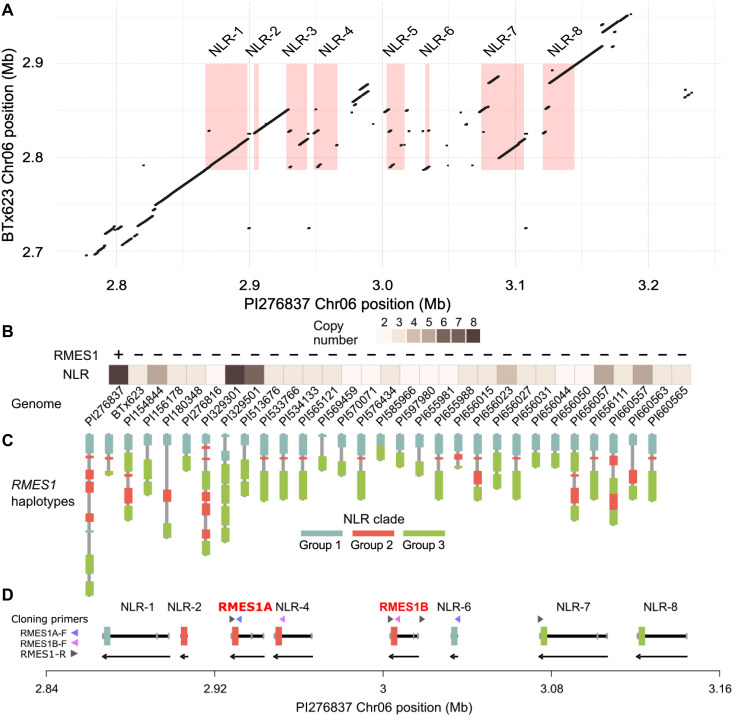
Pangenome structural variant harbors high copy number of NLRs including causal RMES1A and RMES1B. (**A**) Genome alignment of susceptible (BTx623) and resistant (PI276837) haplotypes at *RMES1* encompassing structural variants and NLRs. Red boxes indicate NLR gene locations in PI276837. (**B**) CNV of NLRs at *RMES1* across the sorghum pangenome. (**C**) Pangenome haplotypes of NLR genes at the *RMES1* locus of varying size and genic content. Genes are colored according to phylogenetic clade (fig. S9). (**D**) PI276837 haplotype array of NLRs with identification of *RMES1A* and *RMES1B* from cloning primers (*48*). Coding sequence is colored by clade, 3′ untranslated region and 5′ untranslated region in gray, and intron in black. Arrows below indicate reverse strand orientation.

Given evidence for an NLR mechanism and differences in gene content at the locus, we next investigated the NLR-encoding region across the pangenome. An alignment of BTx623 to PI276837 found a structural variant colocated with the QTL (Fig. 3A). The ~0.2-Mb insertion coincided with both mapping intervals and five NLRs at the locus, accounting for the difference in NLR copy number between BTx623 and PI276837. To understand the extent of *RMES1* NLR copy number variation (CNV), we searched the panproteome for homologous sequences resulting in 163 sorghum homologs and determined their phylogenetic relationship (fig. S9). Homologs were found on Chr06 (*RMES1*) and Chr10. CNV at *RMES1* ranged between two and eight sequences (Fig. 3B). Five clades, established by long well-supported branches, were identified with Chr06 homologs composed of groups 1 to 3 (Fig. 3C and fig. S9). The monophyletic group 1 had low CNV, and only two genomes, including PI276837, contained a second paralog. Group 2 and group 3 clades accounted for the majority of NLR CNV (Fig. 3, B and C).

On the basis of the structural variation coinciding with differential expression (Figs. 1 and 2), we concluded that NLR-1, NLR-3, and NLR-5 were the likeliest candidates to underlie *RMES1* (*47*). During the preparation of this manuscript, two of these NLRs (NLR-3 and NLR-5; named *RMES1A* and *RMES1B*) were reported in the Chinese sorghum variety Henong 16 as causal for *RMES1* aphid resistance (*48*). Using the cloning primers, NLR-3 (SbPI276837.06G016600) and NLR-5 (SbPI276837.06G016800) were identified as *RMES1A* and *RMES1B*, respectively, within the pangenome (Fig. 3D). These genes had been strongly supported by genomic and functional analyses. The Henong 16 haplotype had five tandemly arrayed NLRs, and the causal genes were directly adjacent, indicating that there are at least two resistant haplotypes.

### *RMES1*-encoded NLR proteins have a noncanonical structure

**Fig. 4.**
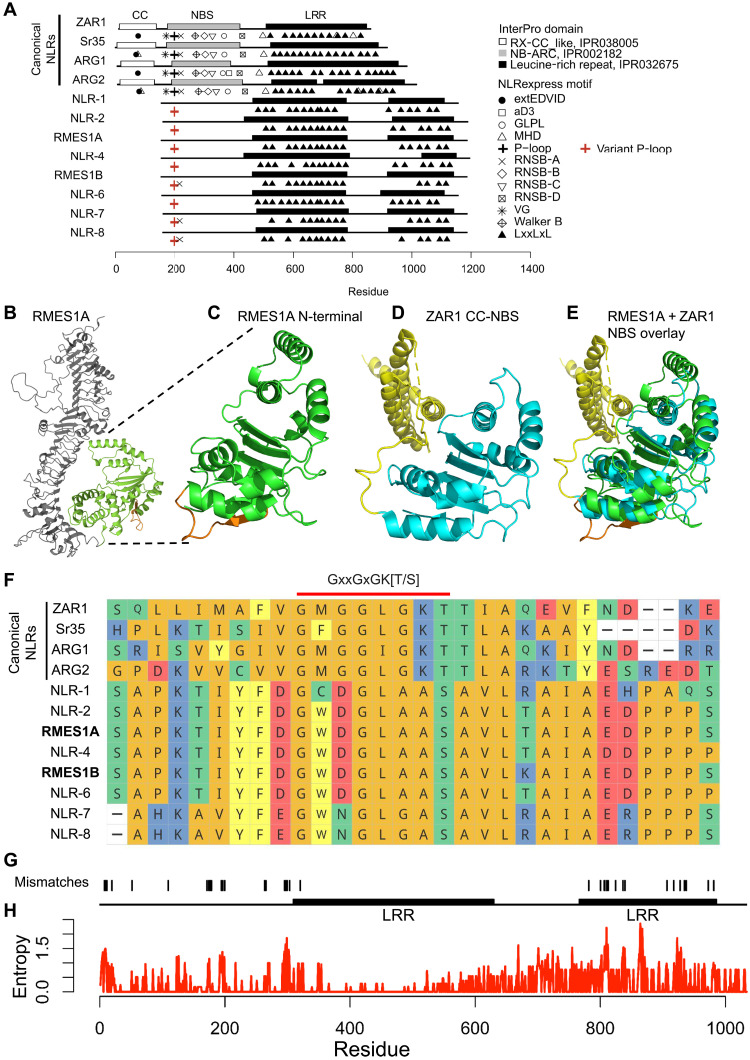
Noncanonical RMES1 NLRs have unusual N-terminal and highly divergent LRR domain. (**A**) Domain and motif annotations of canonical NLRs and noncanonical RMES1 NLRs missing CC and NBS domains. (**B** and **C**) AlphaFold prediction of full length and N-terminal region of RMES1A. LRR region (gray), N-terminal domain (green), and P-loop motif (orange) are colored. (**D**) AlphaFold prediction of ZAR1 CC (gold) and NBS domain (blue). (**E**) Close structural alignment of the RMES1A N-terminal domain (green) to the NBS domain of ZAR1 (blue). (**F**) Sequence alignment of canonical NLR P-loop motifs and RMES1 NLRs. (**G**) Positions of residue mismatches between RMES1A and RMES1B. (**H**) Shannon entropy quantifying highly variable residues across a pangenomic allelic series (group 2). Zero indicates invariable residues.

To better understand the molecular function of the causal NLRs, we investigated their protein structure. Domain and motif prediction identified two LRR regions in all RMES1 paralogs; however, the N terminus did not appear to encode CC or NBS domains typical of *Arabidopsis* and monocot NLRs (Fig. 4A). Structural prediction and similarity analysis with AlphaFold and FoldSeek indicated that the 298 residues at the N terminus of RMES1A and RMES1B were highly similar to the NBS domains of canonical NLRs *Arabidopsis thaliana* RPP13L4 (ZAR1, *E* = 1.5 × 10^−5^) and *Triticum monococcum* CNL9 (Sr35, *E* = 2.8 × 10^−5^) (Fig. 4, B to E, and fig. S10). RMES1A and RMES1B both lack sequence N-terminal to the NBS-like domain; thus, they contain neither the typical CC domain nor a novel domain that could function to activate immunity. The NBS domains of plant NLRs are conserved AAA–adenosine triphosphatase (ATPase) domains that change conformation based on nucleotide binding to a conserved Walker A P-loop motif. The structure of the RMES1 NBS model aligns well with the cryo–electron microscopy structure of ZAR1; however, the P-loop contains sequence variation (GKT > AAS) in RMES1A and RMES1B that is expected to result in a loss of function (Fig. 4F and fig. S11) (*49*). The N-terminal truncation and P-loop substitution are shared across the 163 sorghum homologs (fig. S12). Thus, *RMES1* NLRs, despite being causal for aphid resistance, have two unusual features that are expected to independently result in a loss of function for canonical NLRs.

Sequence conservation between the causal proteins and pangenomic homologs could indicate regions with evolutionary constraints. The PI276837 RMES1A and RMES1B had 96.2% identity (996 of 1035); however, the amino acid residue differences primarily occurred in the NBS-like region or the second LRR domain (Fig. 4G). Phylogenetic clades were used to approximate allelic series, and Shannon’s entropy of residues revealed low and highly variable positions (Fig. 4H). Group 1 had fewer variable residues than groups 2 and 3, which both represented highly variable NLRs (>10 positions with an entropy bit of >1.5) (fig. S13). The second LRR domain had notably higher entropy values than the first. The mutated P-loop motif is conserved across the RMES1 pangenome homologs (entropy = 0). Therefore, it is likely that polymorphisms in the second LRR domain allowed functionalization unrelated to the noncanonical NBS domain.

### Pangenome variation at *RMES1* has ancient evolutionary origin in plants

**Fig. 5.**
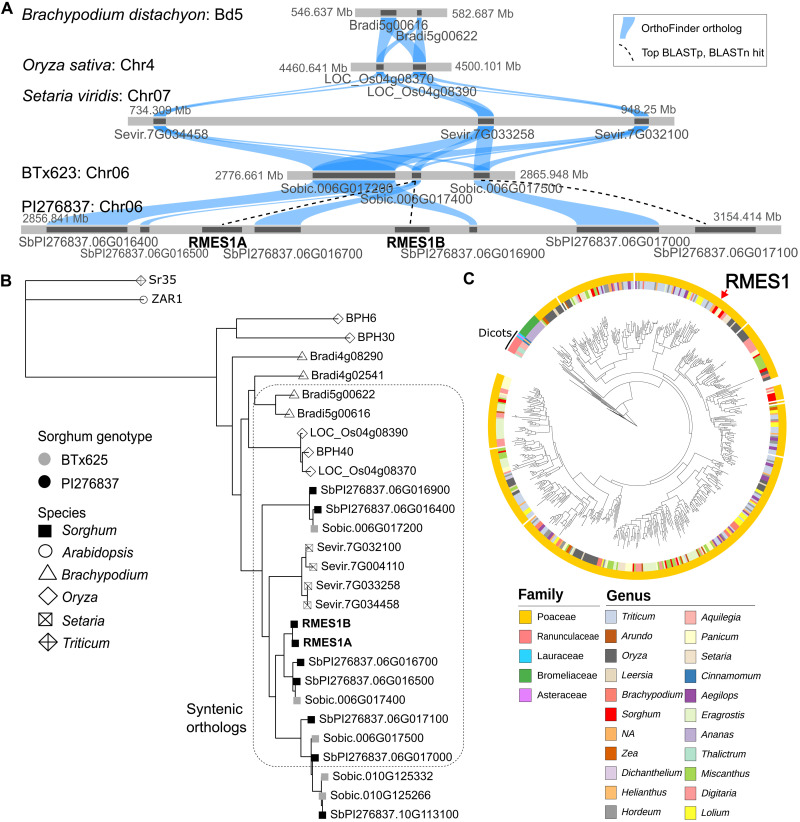
Synteny of RMES1 across the grasses and NLR gene family expansion in plants. (**A**) Syntenic relationships across *B. distachyon* (v3.2), *O. sativa* (v7.0), *S. viridis* (v4.0), and *S. bicolor* (BTx623v5 and PI276837v1) genomes trace the ancient origins of the *RMES1*-related NLR gene family in the grass superpangenome. All genes were within the syntenic orthogroup determined by GeneSpace. Blue connections indicate OrthoFinder orthologs. Dashed lines are best BLASTp and BLASTn hits for orthogroup members. (**B**) Phylogenetic relationships of RMES1 syntenic and global orthologs, noncanonical BPH NLRs, and canonical NLRs. (**C**) Orthologs of *RMES1* across diverse monocot and dicot plant families identified by an HMM for N-terminal sequence.

To trace the origins of *RMES1* beyond the sorghum pangenome, we conducted synteny analysis across the Poaceae superpangenome. The *RMES1* locus was syntenic to Chr05 of *Brachypodium distachyon*, Chr04 of *Oryza sativa*, and Chr07 of *Setaria viridis* (Fig. 5A). At each syntenic locus, two or more NLRs were adjacent to a CAS ortholog. To determine the relationship of RMES1 orthologs across grasses, we built a phylogenetic tree using syntenic and global orthologs from PI276837, BTx623, *Setaria*, *Oryza*, and *Brachypodium*, as well as canonical (*ZAR1* and *Sr35*) and noncanonical NLRs (*BPH6*, *BPH30*, and *BPH40*) (*22*, *23*) (Fig. 5B). The physical and phylogenetically adjacent genes of RMES1 support a tandem duplication, while Chr10 orthologs indicate a segmental duplication originating from Chr06. The brown-planthopper (*Nilaparvata lugens*) resistance gene *BPH40* is a noncanonical NLR allelic to LOC_Os04G08390 and therefore a syntenic ortholog of *RMES1* (*22*). *BPH6* and *BPH30* are more closely related to *RMES1*-like genes than canonical NLRs but are not syntenic to *RMES1*. Poaceae orthologs of *RMES1*, including *BPH40*, share P-loop substitutions (GKT > [A/G]AS) and have truncated N-terminal sequence (fig. S14). To determine whether *RMES1*-like genes occur outside of Poaceae, a Hidden Markov model developed for the RMES1 NBS-like domain identified 556 orthologs found in Poaceae, Bromeliaceae, and the dicot families of Ranunculaceae, Lauraceae, and Asteraceae; however, nearly all (>96%) were found in Poaceae (Fig. 5C).

**Fig. 6.**
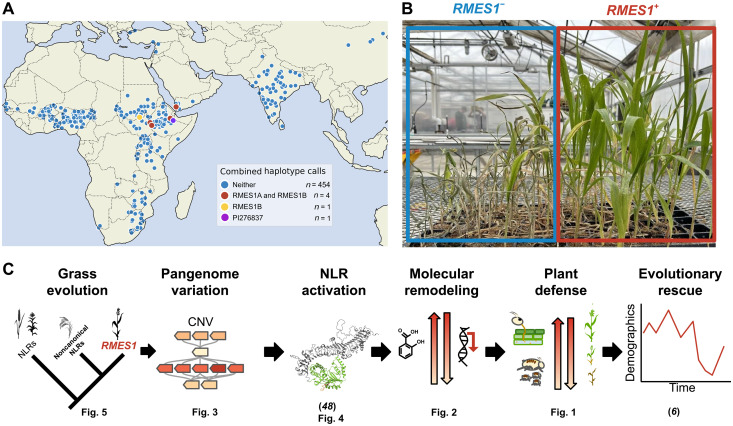
A rare resistant haplotype underlies the genome to phenome map of evolutionary rescue. (**A**) *k*-nucleotide oligomer–based genotyping of *RMES1A* and *RMES1B* in global georeferenced sorghum landrace accessions. (**B**) NILs (NIL^−^, left; NIL^+^, right) after 2 weeks of sorghum aphid infestation. (**C**) Integrated genome-phenome model for the evolutionary rescue of sorghum from sorghum aphid by *RMES1*.

We used a *k*-nucleotide oligomer–based genotyping approach to determining which genotypes of the pangenome resequencing resource had causal RMES1 genes of the PI276837 haplotype. Among 460 landraces across Africa and Asia, only five libraries had both RMES1A and RMES1B and were localized to East Africa and Yemen (Fig. 6A). Among all 2144 resequenced genotypes, 34 libraries had both RMES1 genes including ~10% (13 of 137) in the resequenced subset of the West African Sorghum Association Panel, which represents breeder’s working collections of local landraces and international breeding lines (data S5) (*50*). The *k*-nucleotide oligomer analysis conclusively establishes the rarity of the resistant haplotype in global landraces, which was previously hypothesized on the basis of mid-density sequencing data (*6*), and establishes the presence of *RMES1* in African breeding networks. The Mendelian nature of this noncanonical NLR activation mechanism coupled with the conserved immunity infrastructure of phytohormone signaling and phytoalexin biosynthesis allowed *RMES1* to be rapidly selected for evolutionary rescue (Fig. 6, B and C).

## DISCUSSION

### Ancient gene cluster in the super pangenome leading to recent evolutionary rescue

The genomic basis and molecular mechanisms allowing plant populations to rapidly adapt to changing environmental pressures have not been extensively studied. According to Fisher-Kimura-Orr theory of adaptive walks, early steps of adaptation under changing fitness landscapes are likely to involve large-effect variants (*51*). These variants likely play a larger than expected role in biotic selection where fitness landscapes are more spatially and temporally variable (*52*). NLRs and *R* genes have been known to play important roles in local adaptation to pathogenic pressures; however, their ability to prevent extirpation through rapid selection have not previously been demonstrated (*53*–*55*). Here, we show that a large-effect NLR variant activates common immunity networks to disrupt aphid feeding and was responsible for the evolutionary rescue of sorghum.

Early models of adaptation often focused on de novo mutations, while recent work has supported the prevalence of standing variation (*56*, *57*). Quantitative genetic theory (*58*) and population genomic surveys (*59*) suggest that this standing variation is abundant in most populations and disease resistance QTL often harbors extensive polymorphisms (*60*). Genome organization and expression indicate that *RMES1* arose from a locus undergoing gene birth-and-death and highly polymorphic alleles (*61*) (Figs. 3C and 4H and fig. S13). Neofunctionalization often occurs by mutations to the LRR domain, leading to novel direct or indirect recognition specificities (*62*, *63*). *RMES1* and its cognate effector directly interact at the LRR domain that has high pangenomic variation (Fig. 4H) (*48*). Notably, *M. sorghi* was first reported on Sudanese sorghum (*27*), suggesting that local selection pressure maintained the variant at low frequency in East Africa, providing the standing variation that allowed evolutionary rescue.

### A noncanonical NLR activates conserved immunity networks

The NLR superfamily is capable of triggering immunity with near-limitless inter- and intraspecies diversity (*17*). NLR proteins form resistosomes via oligomerization to activate their N-terminal signaling domains (*16*). By contrast, RMES1 proteins are unusual in that they lack N-terminal domains and contain variation at the NBS P-loop motif predicted to block oligomerization, so they must have a noncanonical function distinct from known NLRs (*49*). Artificial mutations to the catalytic lysine are used in molecular investigations of NLR structure and function; however, natural variants for this residue that are functional for immunity have not been observed. RMES1A and RMES1B may form a functional ATPase and/or signaling mechanism as a heterodimer rescuing conformational activation (*64*).

Similar to *RMES1*, the syntenic brown planthopper resistance gene in *O. sativa* (*BPH40*) appears to require an alternative activation mechanism, indicating that the noncanonical structure-function relationship is conserved (fig. S14) (*22*). However, the heterodimer requirement for function appears to be unique to RMES1, as the BPH40 is individually sufficient for function (*48*). The *Vat* cluster in Cucurbitaceae contains several canonical CC-NLRs; however, the only functional resistance identified at *Vat* is in *Cucumis melo* where *Vat-1* and *PmW* alleles provide resistance to melon aphids and potyviruses or powdery mildew, respectively (*65*). *RMES1* and *BPH40* are notable examples of phloem-feeding resistance across Poaceae, which may be the conserved ancestral function for this gene family or parallel functionalization in *Sorghum* and *Oryza*.

In support of *RMES1* being a noncanonical NLR with immunity functions, there is strong structural similarity to ZAR1 and Sr35 and molecular outcomes similar to NLR activation in response to aphids (Figs. 2 and 4) (*39*, *48*, *66*, *67*). Common outcomes of NLR-activated immunity include phytohormone signaling leading to phytoalexin production, physiological modification, and potentially hypersensitive response (*68*). For aphid immunity, SA is required for the NLR *Mi-1.2* resistance in tomato to *Macrosiphum euphorbiae* and for *RAG1* resistance to soybean aphid, where NLRs are suspected to be causal (*39*, *69*, *70*). Not only did SA rapidly accumulate in *RMES1* NILs, but the PAL pathway also appears to be the dominant biosynthetic route, and a negative ICS response could indicate that it is antagonistic for plant defense (Fig. 2 and fig. S6). The 9-lipoxygenase genes controlling death acid production have been shown to play phytoalexin roles in maize to fungal, lepidopteran, and hemipteran pests (*44*, *71*). The activation of these defense mechanisms by *RMES1* demonstrates that, despite its noncanonical structure and unresolved activation mechanism, the downstream functional outcomes were similar to canonical NLRs (Fig. 2) (*48*).

### Using knowledge of pangenome variation to facilitate evolutionary rescue

An emphasis on pangenomic datasets for crops has seen the number of intraspecific de novo reference genomes rise over the past decade (*72*). As evolutionary rescue relies on de novo or rare variants, it is unlikely that single reference analyses will be sufficient to understand and facilitate this extreme adaptation. Environmental threats, particularly biotic pressures such as insects or pathogens, often require screening extensive diversity to identify resistance (*29*, *73*). Pangenomic references will expedite the identification of adaptive traits that are often absent in first-generation single references (Fig. 3A). Further, pangenomic references will allow efficient identification of trait donors even for extremely rare variants and movement of alleles between the subpopulations existing for global breeding networks (Fig. 6A) (*74*). As crop intensification and ecological disruption continue, it is important to develop agricultural systems that can withstand the emergence of new stressors (*75*). The proliferation and dispersal of many biotic pests will likely lead to more outbreaks requiring rapid adaptation in host species (*76*). One avenue to addressing food instability is the identification and manipulation of the pangenomic basis and molecular mechanisms that can provide evolutionary rescue. The mechanism of *RMES1* as an immunity activation trigger is a valuable example of how plant populations can rapidly adapt to new threats. However, more genome-phenome elucidation of real-world examples of evolutionarily rescued populations will be needed to learn how pangenome knowledge can facilitate adaptation to global change.

## MATERIALS AND METHODS

### NIL development

*RMES1* NILs were developed with a donor parent IRAT204 (*M. sorghi* resistant, donor) and recurrent backcrossing to RTx430 (*M. sorghi* susceptible). Single plant selections were made at the F_2_ generation of each backcross cycle (BC*_x_*F_2_) using the KASP marker for *RMES1* (Sbv3.1_06_02892438R) (*6*), and homozygous +/+ plants were backcrossed. *RMES1*-homozygous BC_3_F_4_ lines (NIL^+^ homozygous for the resistance-linked allele, NIL^−^ homozygous susceptible) were used for all experiments described below.

### Aphid behavior and reproduction assays

*M. sorghi* was received from S. Armstrong at the US Department of Agriculture Agricultural Research Service, Stillwater, OK. Aphids were reared on Tx7000 seedlings under laboratory conditions as previously described (*32*). Seedlings were grown in 4.5″ pots with soil (PRO-MIX BX) and top layer of greens grade (Profile) to reduce damping off. Colonies were grown in a 46 cm–by–46 cm–by–76 cm cage at 60 to 70% relative humidity and a temperature of 24° ± 1°C (BioQuip Products Inc., Rancho Dominguez, CA). For the no-choice assays, single seedlings were grown in 6″ pots. At 3 to 4 weeks of age or GS1 (*77*), three 4- to 5-day-old apterous *M. sorghi* aphids were placed at the base of the seedlings with a camel hair brush. A clear plastic cylinder was placed over the plant to prevent aphids from leaving the pot with an organdy cloth covering for ventilation. The number of aphids on each plant was counted at the same time of day for a week. For the EPG experiments to study aphid feeding behavior, individual age-synchronized aphids (4 to 5 days old) were wired by attaching a thin gold wire (20 μm in diameter) to the dorsum using a conductive silver glue. The wired aphid was gently placed on a 4-week-old sorghum plant. Aphid feeding behavior was recorded for eight consecutive hours using an eight-channel Giga-8 DC-EPG amplifier (*78*). The acquired EPG data were annotated using the Stylet+ software. The discoEPG package was used for calculating feeding parameters and conducting statistical analysis (https://nalamlab.shinyapps.io/test/). The experiment was repeated five times, each including four NIL^−^ and four NIL^+^ plants. Only data with a total of 8 hours of recording were kept for further analysis. A total of 14 NIL^−^ and 13 NIL^+^ plants were used in the analysis.

### Infestation time-course sampling for transcriptomic and metabolomic analysis

A 2 by 3 factorial design was used with NIL^+^ and NIL^−^ plants infested for 24 and 48 HPI and an uninfested (control) sample collected at the same time as the 48-HPI sample. A 50-ml falcon tube with the conical end cut off and a hole with organdy cloth on the cap were placed over the third true leaf of two plants and infested with 20 adult apterous aphids. Cotton balls were used to cover the open end of the tube to ensure maximum response from aphid feeding. Control samples were handled the same way but were not infested. Samples were collected at 12 p.m. after 24 and 48 hours by pooling both leaves. The bottom (basal) inch of tissue from both leaves were flash frozen for sequencing. The remainder of the sample was flash frozen for high-performance liquid chromatography analysis. There were four replicates collected; however, RNA sequencing analysis identified genetic contamination, and six samples were removed from all analyses leaving NIL^+^ control (*n* = 3), NIL^+^ 24 HPI (*n* = 3), NIL^+^ 48 HPI (*n* = 4), NIL^−^ control (*n* = 3), NIL^−^ 24 HPI (*n* = 3), and NIL^−^ 48 HPI (*n* = 2) (fig. S14).

### RNA sequencing and analysis

RNA was extracted using Zymo Quick-RNA minipreps (Thermo Fisher Scientific) and treated with deoxyribonuclease using Invitrogen Turbo DNA-free kit (Thermo Fisher Scientific). RNA quality was checked using a NanoDrop 2000 (Thermo Fisher Scientific), and ~2 μg was submitted on dry ice to Novogene Corporation Inc. (2921 Stockton Blvd., Sacramento, CA 95817, USA), where RNA integrity was confirmed using an Agilent 2100 Bioanalyzer (RIN > 7). Samples were sequenced on an Illumina NovaSeq 6000 Sequencing System with 150-bp paired-end reads with ~54.7 million reads per sample. Trimmed and quality filtered reads generated by Novogene were aligned to the PI276837 (https://phytozome-next.jgi.doe.gov/info/SbicolorPI_276837_v1_1) and RTx430v2 (https://phytozome-next.jgi.doe.gov/info/SbicolorRTx430_v2_1) reference genomes for transcriptome analysis and variant calling (*79*). Reference genomes were indexed and aligned to with STAR v2.7.10 using two-pass mode (*80*). For variant calling, BAM files were processed using the following in GATK v 4.2.5.0, unless otherwise noted: Duplicates were marked and read groups were added using Picard, reads were split using SplitNCigarReads, variants were called individually using HaplotypeCaller, gVCFs were combined using CombineGVCFs, and jointcalled VCF files were produced with GenotypeGVCFs (*81*). Last, VCFs were filtered using VariantFiltration (QUAL > 30, SQR > 3, FS > 60, MQ < 40). VCFs were analyzed in base R v4.4.1 with variants summarized over 0.5-Mb windows, and segregating markers were used to estimate segregation percentage between NILs.

For transcriptome analysis the transcript abundance was quantified using FeatureCounts v2.0.1 (*82*). Differential gene expression was determined using DESeq2 v1.38.1 in R v4.2.2 (*83*, *84*). After removal of contaminated samples, dispersion estimate results suggest that the DESeq2 model assumptions were satisfied (fig. S15). Principal components analysis was performed with R/prcomp and plotted with ggplot2 v3.4.1 (*85*). Coexpression analysis was done using weighted gene coexpression network analysis with the expressed transcriptome (*86*). Coexpression modules were visualized using GGally in ggplot2 (*85*). Genes were determined to be significantly differentially expressed with a Benjamini-Hochberg–adjusted *P* <0.05 and fold change greater than 1.5 (log_2_ fold change > 0.58). Analysis of potential causal gene and pathway candidates were determined a priori through literature search and SorghumBase orthology finder (data S4) (*87*). Genes involved in aphid defense and/or phytohormone signaling in *Arabidopsis* or other species were searched on SorghumBase, and all orthologous genes in BTx623v5 were considered as candidate genes (data S1). Lipoxygenase and jasmonate ZIM-domain gene families were characterized in sorghum previously, and Sobic IDs were already available (*88*, *89*). Global, phytohormone, and defense transcriptome pathways were analyzed using RTx430v2. Transcriptome and genomic analysis of the *RMES1* region was done with PI276837. For analysis of potential causal genes, all 35 PI276837 genes between 2.5 and 3.5 Mb were considered.

### Phytohormone and untargeted metabolome analysis

Phytohormone and metabolome analysis was conducted (on the same samples as transcriptome analysis) following (*90*) with full modifications described in Supplementary Materials and Methods. Briefly, frozen samples were lyophilized, homogenized, and extracted from 30 mg of tissue with 80% methanol. The extracts were analyzed with ultraperformance liquid chromatography–tandem mass spectrometry (UPLC-MS/MS) for general phytohormone profiling and untargeted analysis. Authentic standards used in this assay included JA-d5, JA, SA-D4, SA, IACA, indole-3-carboxylic acid (ICA), indole-2,4,5,6,7-d5-3-acetic acid (IAA-D5), 12-OPDA, IA-aspartic acid (IA-Asp), ABA-D6, l-alanine-^13^C_3_, l-phenylalanine-^13^C_6_, fumaric acid–^13^C_4_, l-tryptophan-^13^C_11_, and indole-3-acetic acid-^13^C_6_.

Phytohormones that were detected and quantified include SA, JA, ABA, 12-OPDA, IAA, ICA, IACA, and IA-Asp. UPLC-MS/MS analysis was performed on a Waters ACQUITY Classic UPLC Premier T3 1.7 μm coupled to a Waters Xevo TQ-S triple quadrupole mass spectrometer. Raw data were analyzed with Skyline v21 open-source software for retention time and peak area integration (*91*). Peak areas were extracted for target compounds detected in biological samples and normalized to the peak area of the appropriate internal standard or surrogate in each sample. Absolute quantitation (in nanograms per gram) was calculated using the linear regression equation generated for each compound from the calibration curve.

Untargeted metabolomes were quantified on a Waters Acquity UPLC system. Separation was achieved using a Waters ACQUITY UPLC Premier T3 1.7 μm Column, using a gradient from solvent A (0.1% formic acid in water) to solvent B (0.1% formic acid in acetonitrile) and a flow rate of 0.5 ml/min. The column eluent was infused into a Waters Xevo G2-XS quadrupole orthogonal acceleration–time-of-flight MS with an electrospray source in negative ionization sensitivity mode, with MS^E^ data–independent MS/MS acquisition. XCMS (*92*, *93*) version 3.20.0 was used to process raw data using R v4.2.2. RAMClustR version 1.2.4 in R version 4.2.2 was used to normalize, filter, and group features into spectra (*94*). MSFinder (*95*) was used for spectral matching, formula inference, and tentative structure assignment. Results were imported into the RAMClustR object. A total score was calculated on the basis of the product scores from the findmain function and the MSfinder formula and structure scores. A total of 14,130 annotation hypotheses were tested for 4306 compounds. The highest total score was selected for each compound, considering all hypotheses. Direct parent class was used in the manuscript, and full annotation is reported in data S2. For statistical analysis of phytohormones, a two-way ANOVA was performed, fitting a linear model with genotype, time point, and their interaction. Pairwise statistical comparisons of metabolite abundance were conducted using Welch’s *t* test (two-sided, unequal variance) in R. Fold change was calculated as the ratio of group means, and Benjamini-Hochberg–adjusted *P* values were reported.

### Comparative genomics analyses

Intraspecific orthology of *RMES1-*like NLRs was determined using the sorghum panproteome (https://phytozome-next.jgi.doe.gov/sorghumpan/). Sequences were clustered using MMseqs2 (*96*) using the following parameters: minimal sequence identity 50%, coverage 80%, coverage mode 1 (--min-seq-id 0.5 -c 0.8 --cov-mode 1). All *RMES1* NLR candidates were found in a single cluster. Full-length cluster sequences were aligned using mafft v7.505 (*97*) with default parameters, and cluster phylogeny was constructed using RAxML-NG (*98*) with the following options: model JTT, 100 bootstrap replicates (raxml-ng --all --bs-trees 100 --model JTT). The resulting tree had five well-supported (bootstrap > 95%) clades corresponding to group 1, group 2, and group 3 sequences on Chr06 and group 4 and group 5 sequences on Chr10. We extracted the corresponding subalignments and calculated per-position Shannon entropy as previously described (*99*).

Orthology of sorghum genes across Poaceae was determined using GENESPACE with default specifications (*100*). Briefly, GENESPACE applies OrthoFinder (*101*) to the protein sequences of 30 sorghum pangenome members, *Setaria*, *Brachypodium*, and *Oryza* and then parses the blast hits to syntenic regions. The resulting syntenic and phylogenetically hierarchically orthologous sets of genes [“orthogroups” (OGs)] were used for phylogenetic comparisons. CNV was identified as genes belonging to the same OG and shared in both BTx623v5 and PI276837 but at different copy numbers (*102*). Syntenic orthologs from GENESPACE were then used in “riparian” plots to track their positions across genomes. To determine gene locations within repeat expansions, we mapped 100-bp windows with 50-bp overlaps from query to reference target sequences using DEEPSPACE (github.com/jtlovell/DEEPSPACE), which calls minimap2 on windowed sequences. The resulting dot plots can be integrated with positional coordinates of genes to understand broader patterns of sequence expansion. The presence of *RMES1*-like genes outside of *Poaceae* was determined with an hidden Markov model (HMM) of the noncanonical N terminus. The original cluster alignment was reduced manually using jalview to remove gappy columns and gappy sequences and was trimmed to correspond to the N-terminal NB-ARC–like domain and an HMM was produced. This was used to search the UniRef90 database with SeqKit (*103*).

### Protein motif/domain analyses and structural modeling

Protein sequences for PI276837 RMES1 NLRs, Sobic.007G085400 (*ARG1*), and Sobic.005G047700 (*ARG2*) as well as canonical AtZAR1 (AEE78731.1) and TaSr35 (AGP75918.1) were used to determine motif and domain presence with NLRexpress and InterPro v101.0 (*104**,* *105*). We used AlphaFold2 (*106*) as implemented in ColabFold (*107*) to predict the structures of NLR candidates at *RMES1*. Structural homologs of the N-terminal non-LRR domain of *RMES1* NLR candidates were identified using Foldseek (*66*). Structures were visualized in Chimera (*108*).

### *k*-nucleotide oligomer genotyping

*k*-nucleotide oligomer genotyping of the *RMES1* locus from *S. bicolor* reference PI276837 was conducted within Illumina resequencing libraries (*n* = 2143). Single-copy 80-bp *k*-nucleotide oligomers (80mers) were extracted from genes SbPI276837.06G016600 and SbPI276837.06G016800, generating 15,507 and 13,507 80mers, respectively. To ensure that these 80mers were (i) unique to PI276837 and (ii) could be genotyped in short-read Illumina data (i.e., “typable” markers), single-copy 80mers were searched using exact matches in Illumina polishing libraries for each pangenome reference. Only 80mers present in PI276837 and absent in all other references with a minimum observed count of one and a maximum observed count of 100 were retained. This search yielded 3586 typable 80mers, 1322 for SbPI276837.06G016600 and 2264 for SbPI276837.06G016800. Next, the typeable markers were used to genotype all resequenced individuals using the same exact match criteria as the Illumina reference polishing libraries. On the basis of the histogram distribution of the number of *k*-nucleotide oligomer matches per gene, genes with more than 95% of total typable matches were considered present within that library.
